# Excessive splenic volume is an unfavorable prognostic factor in patients with non-small cell lung cancer treated with chemoradiotherapy

**DOI:** 10.1097/MD.0000000000023321

**Published:** 2020-12-04

**Authors:** Jianping Guo, Lei Wang, Xiaoyan Wang, Luo Li, Yajuan Lü, Congcong Wang, Chong Hao, Jiandong Zhang

**Affiliations:** a Department of Radiotherapy, Shandong Provincial Qianfoshan Hospital, Shandong University, Jinan; b Department of Oncology, Maternal and Child Health Care Hospital of Zibo; c Department of Oncology, The Fourth People's Hospital of ZiBo City; d Department of Science and Education, Zibo Central Hospital, Zibo, Shandong, China.

**Keywords:** chemoradiotherapy, non-small cell lung cancer, prognostic factor, splenic volume

## Abstract

The relationship between splenic volume and the outcome of chemoradiotherapy for lung cancer has rarely been studied or addressed. The purpose of our study was to investigate whether splenic volume was associated with prognosis in patients treated with chemoradiotherapy for advanced or locally advanced non-small cell lung cancer (NSCLC).

A retrospective investigation was conducted. Finally, 202 patients met the criteria and were included in the study. All patients were divided into 2 groups according to the optimum cutoff value of splenic volume for overall survival (OS). The optimum cutoff value was identified by X-tile software, and the OS and disease-free survival (DFS) were compared between the 2 groups of patients. The impact of splenic volume and other clinical characteristics on OS and DFS was analyzed using the Kaplan–Meier method and Cox proportional hazards model. Clinical characteristics were compared using chi-square or Fisher exact tests.

The median (range) of splenic volume was 156.03 (28.55–828.11) cm^3^. The optimal cutoff value of splenic volume was 288.4 cm^3^. For univariate analyses, high splenic volume was associated with decreased OS (*P* = .025) and DFS (*P* = .044). In multivariate analyses, splenic volume remained an independent predictor of OS as a binary dependent variable (*P* = .003).

Excessive splenic volume was associated with decreased OS and DFS in patients with NSCLC treated with chemoradiotherapy. Splenic volume should be regarded as an independent prognostic factor for patients treated with chemoradiotherapy for advanced or locally advanced NSCLC.

## 1 Introduction

Lung cancer is the most common form of cancer worldwide and is the leading cause of cancer-related mortality.^[1]^ The spleen is an organ with a unique anatomical structure and cellular composition. It plays an important role in immune surveillance and response. However, our understanding of the relationship between the spleen and the pathogenesis of the disease is insufficient. The spleen contains a large number of immune-related cells such as lymphocytes and macrophages that play an important role in the immune function of the spleen.^[2]^ As the largest secondary lymphoid organ in the body, many studies to identify spleen cell subpopulations, locations, and functions has been performed in mice, although comparisons with humans remain to be clarified.^[3]^

Although the immune function of the spleen is not easily evaluated, there is a close relationship between splenic volume and splenic function.^[4]^ In daily diagnosis and treatment work, we found that there were great discrepancies in splenic volume for patients with non-small cell lung cancer (NSCLC). The spleen contains a large number of immune cells that could affect the prognosis of many diseases.^[5]^ The relationship between splenic volume and the outcome of lung cancer has rarely been studied or addressed. Therefore, we performed a retrospective study to investigate the relationship between splenic volume and the outcome of patients with advanced or locally advanced NSCLC treated with chemoradiotherapy.

## 2 Materials and methods

### 2.1 Study design and enrolled patients

A retrospective analysis was used in this study, and we surveyed 347 patients with advanced or locally advanced NSCLC. There were 202 patients who were treated at the Shandong Provincial Qianfoshan Hospital in Jinan and the Maternal and Child Health Care Hospital of Zibo (China) between January 2014 and August 2016, and they met the criteria. Chemoradiotherapy was used as the initial treatment. Pathological results were obtained by bronchoscopy or puncture biopsy, and epidermal growth factor receptor (EGFR) mutational status was obtained by genetic testing of the patients. The standard clinical staging of the patients was determined by physical examination, positron emission tomography (PET), or whole-body enhanced computed tomography (CT) scan. All patients who were unable or reluctant to undergo surgical treatment were treated with chemoradiotherapy as the initial therapy according to the practice. Patients who were excluded from the study were those with small cell types, those who were uncooperative or inaccessible, and those from whom complete information could not be obtained. In order to eliminate the influence of splenomegaly caused by cirrhosis in this study, all patients had been excluded history of cirrhosis or hypersplenism. All patients signed the informed consent approved by the Institutional Committee of the Shandong Provincial Qianfoshan Hospital and the Maternal and Child Health Care Hospital of Zibo on Human Rights in Research.

## 3 Data collection

### 3.1 Splenic volume

CT scans were performed before administration of radiotherapy to the patients. Then, the CT image was transmitted to the radiotherapy treatment planning system (Eclipse, America). The contours of the spleen were plotted on a computer by the same physician, and the volume of the spleen was calculated using the radiotherapy treatment planning system (Eclipse, America).

### 3.2 Clinical characteristics

Clinical characteristics were collected and included age, sex, smoking status, histology, clinical stage, epidermal growth factor receptor(EGFR) mutational status, chronic obstructive pulmonary disease (COPD) concomitant state, absolute neutrophil count(ANC), absolute lymphocyte count (ALC), absolute monocyte count (AMC), red blood cell count (RBC), and platelet count (PLT).

### 3.3 Follow-up

Follow-up data were collected until death or for 36 months. All patients were regularly followed up. We collected material, including the results of laboratory and imaging examinations every 3 months in the first year, every 6 months in the second year, and annually thereafter.

### 3.4 Statistical analysis

To analyze the predictive value of the splenic volume for overall survival (OS) and disease-free survival (DFS) in patients with advanced or locally advanced NSCLC, we selected X-tile software to separate all patients into 1 of 2 groups according to splenic volume. This software can be utilized to define an optimum cutoff point for the numerical variables required to predict prognosis. In our study, the cutoff point for splenic volume was defined according to the OS. The optimum cutoff points for the ANC, ALC, AMC, RBC, and PLT were also identified by X-tile.

The differences in clinical characteristics between the 2 groups were assessed using the chi-squared test or Fisher exact test for categorical variables. OS was defined as the time from the initial treatment until death for any reason, while DFS was defined as the time from the initial treatment until disease progression or death for any reason. The Kaplan–Meier method was used to estimate the survival probabilities using GraphPad Prism 7.0 (GraphPad Software). The log-rank test was used to statistically compare the curves of the 2 groups. In univariate and multivariate analyses, the Cox proportional hazard model was used to determine the hazard ratio (HR) of variables to OS and DFS, and the data were analyzed using the statistical package SPSS version 25.0 (SPSS Inc., Chicago, IL). The results are expressed as hazard ratios (HRs) with their 95% confidence intervals (CIs). A 2-sided *P*-value < .05 was considered statistically significant.

## 4 Results

### 4.1 Patient characteristics

A total of 202 patients participated in our study. The clinical characteristics of the patients are shown in Table 1.

**Table 1:** Patient characteristics based on the splenic volume.

	Splenic volume		
	Low (<288.4 cm^3^)	High (≥288.4 cm^3^)	*P* value
Age			
<65 years	104	19	.278
≥65 years	71	8	
Gender			
Male	143	22	.997
Female	32	5	
Smoking status			
Never	61	12	.334
Current/former	114	15	
Histology			
Adenocarcinoma	82	13	.900
Other	93	14	
Clinical stage			
II–III	79	8	.130
IV	96	19	
EGFR status			
Mutant type	44	7	.931
Wild type	131	20	
COPD			
Yes	47	8	.763
No	128	19	
ANC (×10^9^/L):			
<6.7	141	26	.083
≥6.7	34	1	
ALC (×10^9^/L):			
<1.2	51	3	.049
≥1.2	124	24	
AMC (×10^9^/L):			
<0.5	112	24	.019
≥0.5	63	3	
RBC (×10^9^/L):			
<4.8	134	23	1.000
≥4.8	20	4	
PLT (×10^9^/L):			
<232	85	23	.001
≥232	90	4	

ALC = absolute lymphocyte count; AMC = absolute monocyte count; ANC = absolute neutrophil count; COPD = chronic obstructive pulmonary disease; EGFR = epidermal growth factor receptor; RBC = red blood cell count; PLT = platelet count.

### 4.2 Determination of cutoff values for splenic volume, ANC, ALC, AMC, RBC, and PLT

**Figure 1:**
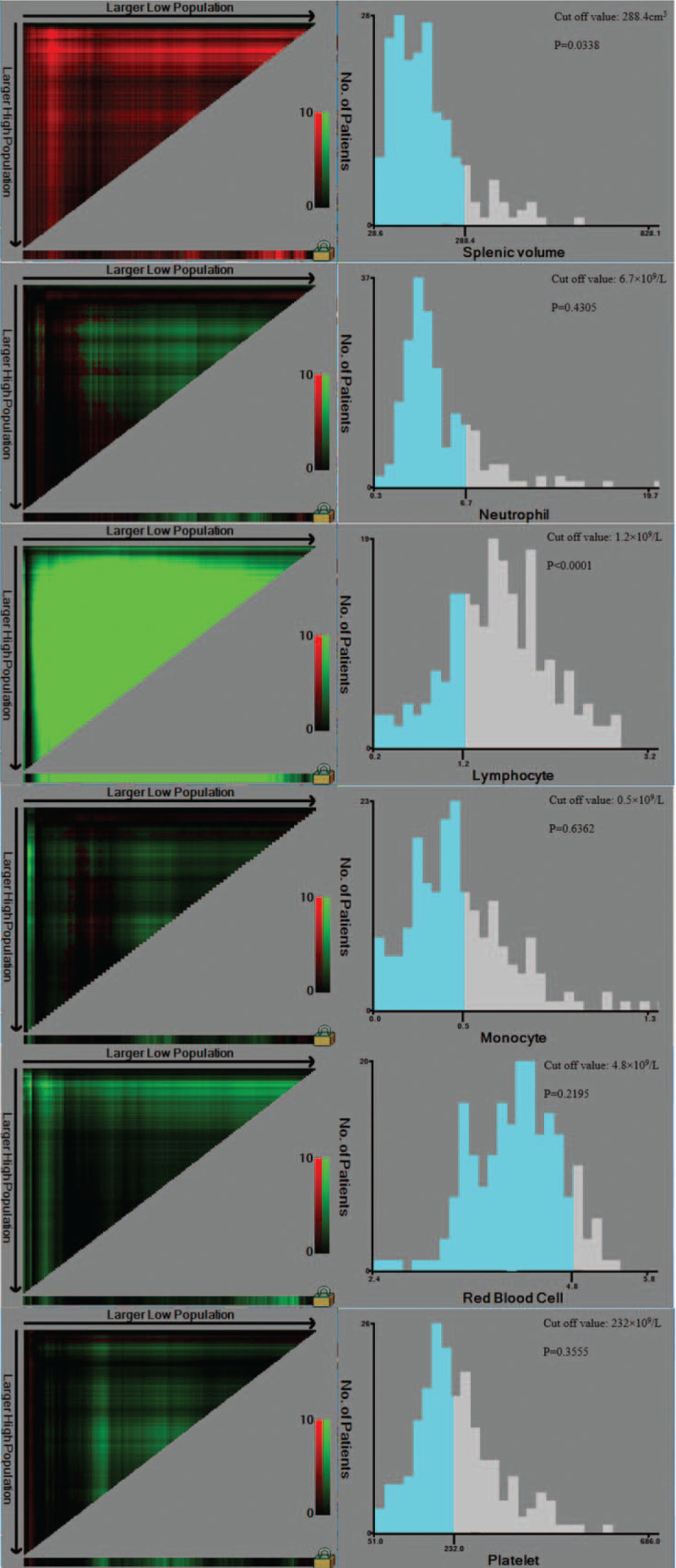
Calculation of cut off value for splenic volume, absolute neutrophil count (ANC), absolute lymphocyte count (ALC), absolute monocyte count (AMC), red blood cell count (RBC), and platelet count (PLT) for overall survival (OS) by X-tile software.

The optimum values for splenic volume, ANC, ALC, AMC, RBC, and PLT for OS in patients with advanced or locally advanced NSCLC treated with chemoradiotherapy were selected using X-tile software. This software can be utilized to define the optimum cutoff point of a numerical variable required to predict prognosis. For OS, and using a spleen volume of 288.4 cm^3^ as the cutoff value, the minimum *P* value (.0338) can be obtained. Thus, we utilized a splenic volume of 288.4 cm^3^ as the optimum cutoff value. The patients were divided into the low splenic volume (<288.4 cm^3^; n = 175 [86.6%]) group and high splenic volume (≥288.4 cm^3^; n = 27 [23.4%]) group. For OS, the optimum cutoff value was 6.7 × 10^9^/L for ANC (*P* = .4305), 1.2 × 10^9^/L for ALC (*P* < .0001), 0.5 × 10^9^/L for AMC (*P* = .6362), 4.8 × 10^9^/L for RBC (*P* = .2195), and 232 × 10^9^/L for PLT (*P* = .3555), respectively. The optimum cutoff values are shown in Fig. 1 for splenic volume, ANC, ALC, AMC, RBC, and PLT for the prognosis of patients with advanced or locally advanced NSCLC treated with chemoradiotherapy.

### 4.3 OS and DFS according to splenic volume

**Figure 2:**
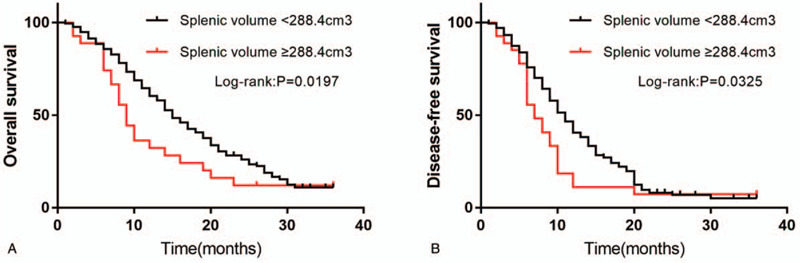
(A) Kaplan–Meier curves for overall survival (OS) according to splenic volume. (B) Kaplan–Meier curves for disease-free survival (DFS) according to splenic volume.

As shown in Fig. 2, using the Kaplan–Meier method for analysis, patients with high splenic volume exhibited significantly shorter OS than those with low splenic volume (*P* = .0197). The median time and 3-year OS rate were 9 months and 48.5% in the high splenic volume group, and 15 months and 44.4% in the low splenic volume group, respectively. The DFS in patients with high splenic volume was also significantly poorer than that in those with low splenic volume using the same method (*P* = .0325). The median time and 3- year DFS rates were 7 months and 77.8% in the high splenic volume group, and 11 months and 46.6% in the low splenic volume group, respectively.

### 4.4 Univariate and multivariate analyses for OS and DFS

**Figure 3:**
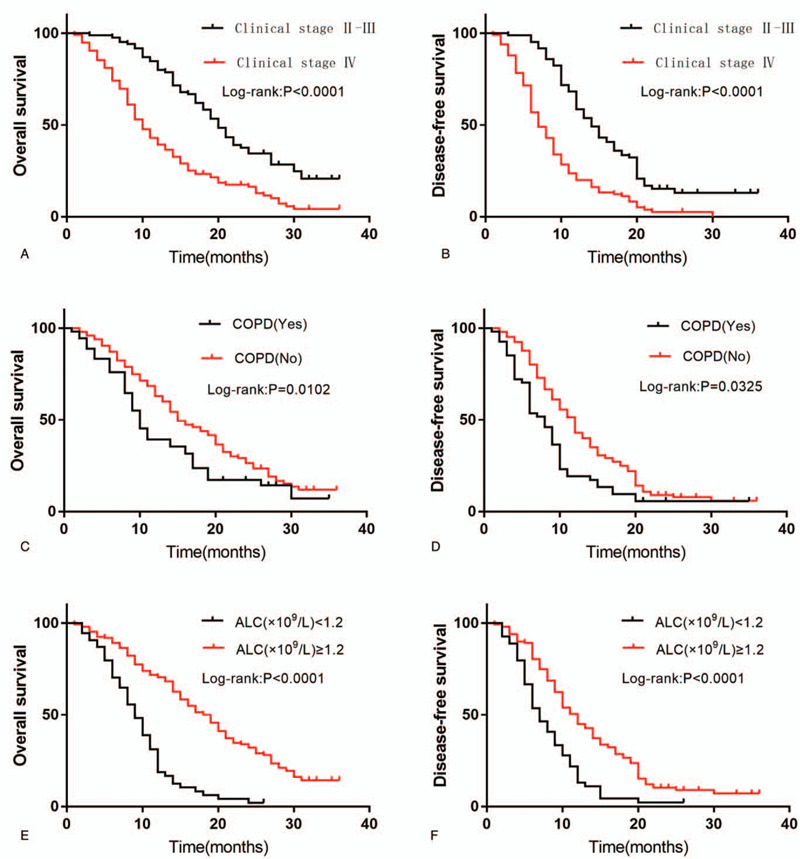
(A) Kaplan–Meier curves for overall survival (OS) according to clinical stage. (B) Kaplan–Meier curves for disease-free survival (DFS) according to clinical stage. (C) Kaplan–Meier curves for OS according to concomitant state of chronic obstructive pulmonary disease (COPD). (D) Kaplan–Meier curves for DFS according to concomitant state of COPD. (E) Kaplan–Meier curves for OS according to absolute lymphocyte count (ALC). (F) Kaplan–Meier curves for DFS according to ALC.

Using the Cox proportional hazard model, the univariate analysis revealed that splenic volume (HR: 1.664, 95% CI: 1.067–2.594, *P* = .025), clinical stage (HR: 2.418, 95% CI: 1.747–3.347, *P* = .000), COPD concomitant state (HR: 1.566, 95% CI: 1.107–2.217, *P* = .011), and ALC (HR: 0.300, 95% CI: 0.209–0.430, *P* = .000) were significantly associated with OS. Splenic volume (HR: 1.548, 95% CI: 1.011–2.370, *P* = .044), clinical stage (HR: 2.500, 95% CI: 1.842–3.394, *P* = .000), and ALC (HR: 0.461, 95% CI: 0.330–0.645, *P* = .000) were significantly associated with the DFS. Table 2 shows the outcomes of the univariate analysis. Multivariate analysis showed that splenic volume (HR: 0.492, 95% CI: 0.307–0.789, *P* = .003), clinical stage(HR: 2.380, 95% CI: 1.659–3.415, *P* = .000), COPD concomitant state (HR: 0.609, 95% CI: 0.422–0.879, *P* = .008), and ALC (HR: 0.328, 95% CI: 0.221–0.487, *P* = .000) were significantly associated with OS. Clinical stage (HR: 2.575, 95% CI: 1.837–3.610, *P* = .000), COPD concomitant state (HR: 0.547, 95% CI: 0.386–0.776, *P* = .001), and ALC (HR: 0.520, 95% CI: 0.361–0.748, *P* = .000) were significantly associated with the DFS. Table 3 presents the outcomes of the multivariate analysis. The OS and DFS for patients according to clinical stage, COPD concomitant state, and ALC using the Kaplan–Meier method are shown in Fig. 3.

**Table 2:** Univariate analyses of factors associated with overall survival and disease-free survival.

	Disease-free survival		Overall survival	
Variable	Hazard ratio (95%CI)	*P* value	Hazard ratio (95%CI)	*P* value
Age				
<65 vs ≥65	0.923 (0.687–1.241)	.596	0.985 (0.720–1.346)	.923
Gender				
Male vs female	1.194 (0.810–1.759)	.371	1.209 (0.806–1.813)	.359
Smoking status				
Never vs current/former	0.986 (0.727–1.338)	.928	1.027 (0.471–1.422)	.874
Histology				
Adenocarcinoma vs otner	0.915 (0.682–1.227)	.552	0.968 (0.709–1.323)	.840
Clinical stage				
II–III vs IV	2.500 (1.842–3.394)	.000	2.418 (1.747–3.347)	.000
EGFR status				
Mutant type vs Wild type	1.711 (0.910–3.232)	.096	1.704 (0.894–3.272)	.110
COPD				
Yes vs No	0.150 (0.020–1.098)	.062	1.566 (1.107–2.217)	.011
ANC (×10^9^/L)				
<6.7 vs ≥6.7	0.759 (0.524–1.100)	.146	0.662 (0.437–1.002)	.051
ALC (×10^9^/L)				
<1.2 vs ≥1.2	0.461 (0.330–0.645)	.000	0.300 (0.209–0.430)	.000
AMC (×10^9^/L)				
<0.5 vs ≥0.5	1.034 (0.759–1.409)	.831	0.918 (0.660–1.278)	.614
RBC (×10^9^/L)				
<4.8 vs ≥4.8	0.712 (0.451–1.124)	.145	0.630 (0.380–1.043)	.073
PLT (×10^9^/L)				
<232 vs ≥232	0.780 (0.581–1.048)	.099	0.736 (0.538–1.006)	.055
Splenic volume				
<288.4 cm^3^ vs ≥288.4 cm^3^	1.548 (1.011–2.370)	.044	1.664 (1.067–2.594)	.025

ALC = absolute lymphocyte count, AMC = absolute monocyte count, ANC = absolute neutrophil count, COPD = chronic obstructive pulmonary disease, EGFR = epidermal growth factor receptor, PLT = platelet count, RBC = red blood cell count.

**Table 3:** Multivariate analyses of factors associated with overall survival and disease-free survival.

	Disease-free survival		Overall survival	
Variable	Hazard ratio (95%CI)	*P* value	Hazard ratio (95%CI)	*P* value
Clinical stage				
II–III vs IV	2.575 (1.837–3.610)	.000	2.380 (1.659–3.415)	.000
COPD				
Yes vs No	0.547 (0.386–0.776)	.001	0.609 (0.422–0.879)	.008
ALC (×10^9^/L)				
<1.2 vs ≥1.2	0.520 (0.361–0.748)	.000	0.328 (0.221–0.487)	.000
Splenic volume				
<288.4 cm^3^ vs ≥288.4 cm^3^	0.759 (0.524–1.100)	.146	0.492 (0.307–0.789)	.003

ALC = absolute lymphocyte count, COPD = chronic obstructive pulmonary disease.

## 5 Discussion

In the present study, our main purpose was to examine the relationship between splenic volume and outcomes of patients with advanced or locally advanced NSCLC treated with chemoradiotherapy. Using the Kaplan–Meier method, significant differences in OS and DFS between groups with different splenic volumes were found. Moreover, univariate and multivariate analyses for OS and DFS using the Cox proportional hazard model also confirmed that the splenic volume was an independent risk factor for patients with advanced or locally advanced NSCLC treated with chemoradiotherapy.

It is difficult to determine the functional status of the immune system in different species. The optimal immunologic defense is not necessarily the maximum immunologic defense due to the risk of autoimmunity.^[6]^ As the largest lymphoid organ of the human body, the spleen is rich in immune cells and has a unique anatomical structure. It monitors all circulating blood components and can optimally phagocytize any of them, thus playing an important role in defending against pathogens. The function of the spleen is difficult to quantify. In recent years, several methods have been studied to quantify the many different functions of the spleen. However, the exact function of the spleen remains difficult to evaluate.^[7]^ The spleen is a multifunctional organ with a unique structure and cellular composition. The circulatory system, reticuloendothelial system, and immune system interact through the spleen.^[2]^

In clinical work, we found that there was a great discrepancy in the splenic volume for patients with NSCLC and many patients with splenomegaly. The underlying mechanism is unknown. Splenomegaly is often seen in patients with hepatocellular carcinoma and cirrhosis, and a strong correlation between spleen size and hepatocellular carcinoma and cirrhosis has been reported in some studies. Increased portal venous system resistance is considered the most important cause of splenomegaly.^[8]^ In addition, other factors may lead to splenomegaly in patients, such as splenic metastasis of carcinoma, splenic aneurysm, splenic tuberculosis, or splenic hemangioma.^[9–12]^ For patients with tumors, whether there are other mechanisms that cause splenomegaly requires further study.

A possible mechanism for splenomegaly in patients with tumors was found through a mouse experimental model. Fang et al^[13]^ found that disorders of T-lymphocyte circulation in the spleen caused by dysfunction of β-actin and S100-A9 protein expression and an increase in the quantity of splenocytes were the reasons for the occurrence of splenomegaly in hepatocellular carcinoma-bearing mice.

In our study, there was a significant difference in ALC between the 2 groups with different splenic volumes, as shown in Table 1. This difference may be due to the large number of lymphocytes trapped in the spleen. Therefore, we considered that this hypothesis may also be one of the mechanisms leading to splenomegaly in some patients with NSCLC. Large numbers of lymphocytes accumulate in the spleen in patients with cancer, and lymphocytes are highly sensitive to radiation. Low-dose radiation can decrease the number of peripheral blood lymphocytes.^[14]^ The spleen is also a lymphoid organ, and radiotherapy-related lymphopenia also occurs after irradiation of the spleen.^[15]^ Previous studies have claimed that higher spleen dose–volume parameters are associated with severe lymphopenia during chemoradiotherapy for esophageal cancer.^[16]^ Whether it is necessary to protect the spleen from receiving an excessive radiation dose during radiotherapy is a question worth considering. Whether it is necessary to protect the spleen from receiving an excessive radiation dose during radiotherapy is a question worth considering.

The mechanism of splenomegaly as a poor prognostic factor in patients with NSCLC deserves further investigation. Previous studies have found that although the spleen contains a large number of lymphocytes, these lymphocytes are mainly composed of inhibitor precursor and inhibitor/inducer T cells, and these cells gradually mature during the migration from the spleen but do not play an active role in immune function. During the development of cancer, the spleen does not contribute to positive immune function, but rather plays a negative role.^[17,18]^ We considered that increased production of inhibitor T cells may be the reason that splenomegaly indicates a poor prognosis. Many studies have reported that high levels of CD4+T cells indicate strong immune function, and high levels of CD8+T cells indicate reduced immune function. In addition, immune function is also affected by the ratio of CD4+/CD8+ T cells.^[19–21]^ A change in immune function is one of the factors affecting prognosis.^[22]^ Changes in the ratio of CD4+/CD8+ T cells were confirmed by experiments with carcinoma-bearing mice.^[13]^ Consequently, a decreased ratio of CD4+/CD8+ T cells should also be considered as a possible reason why splenomegaly indicates poor prognosis.

The effects of splenic irradiation on the body of cancer patients are still not clear. Some studies have suggested that splenic irradiation can cause radiation-related lymphopenia and damage the immune function, which adversely affects the body.^[23,24]^ However, some studies have indicated that radiation-related lymphopenia does not affect the overall survival rate of patients.^[17]^ Some studies suggest that tumor irradiation combined with spleen irradiation can result in additional T cell aggregation in the tumor microenvironment, which assists with controlling tumors.^[25]^ A larger spleen contains more lymphocytes, and our view also is that splenic irradiation will not adversely affect patients.

Several studies have indicated that an oncogenic change causes an inflammatory microenvironment. Inflammation in the tumor microenvironment enables the proliferation and survival of malignant cells, promotes angiogenesis and metastasis, destroys the adaptive immune response, and changes the response to hormones and chemotherapy drugs. Tumor-associated macrophages (TAMs) can sustain the inflammatory microenvironment and participate in carcinogenesis and/or tumor invasion and metastasis.^[26–29]^ The functions of TAMs include support of tumor-related angiogenesis, promotion of tumor cell invasion, migration, perfusion, and suppression of anti-tumor immune responses.^[30]^ TAMs exist in several cancer types, including breast, lung adenocarcinoma, and Hodgkin lymphoma, and correlate not only with increased vascular density but also with a worse clinical outcome.^[31–33]^

Tumor-associated neutrophils (TANs) are also important inflammatory cells in the tumor microenvironment. A meta-analysis revealed that TANs are a poor prognostic indicator of survival in a variety of malignancies, and the underlying mechanism of their role in promoting and developing cancer has only been partially elucidated. The mechanism includes the promotion of proliferation and survival of malignant cells, the diversion of adaptive immunity, and the promotion of extracellular matrix remodeling, invasion, angiogenesis, and lymphangiogenesis.^[28]^ TAMs and TANs are the major components of myeloid-derived suppressor cells capable of promoting tumor development. TAMs are replenished faster than TANs in tumor-bearing mice.^[34,35]^

Cortez-Retamozo et al^[36]^ performed animal studies in a conditional genetic mouse model of lung adenocarcinoma and found that the spleen is an important source of TAMs and TANs. The spleen can continuously supply growing tumors with these cells. A large number of TAM and TAN precursors actually relocate from the spleen to the tumor stroma, and resection of the spleen before or after tumor development can significantly reduce TAM and TAN responses and delay tumor growth. These researchers concluded that the spleen contributes to TAMs and TANs and contributes to tumor growth. Studies show that the spleens of rodents are slightly different on an anatomical level when compared with those of humans.^[5]^ We suspect that splenomegaly may affect the prognosis of patients with NSCLC through increased TAMs and TANs. This hypothesis also supports the idea that splenic irradiation does not adversely affect patients. In contrast, damage to the spleen due to radiation is beneficial to patients with NSCLC because it invokes a heightened immune response. This is different from some views and is worthy of further exploration. The current study did not include the average dose of splenic irradiation received by patients, and it is likely that the dose–volume of the spleen may affect the survival of patients. The effect of splenic irradiation on patients requires further study.

## 6 Conclusions

In conclusion, our study demonstrated that splenic volume is a prognostic factor in patients with advanced or locally advanced NSCLC who receive chemoradiotherapy. Excessive splenic volume was associated with shorter OS and DFS. We discussed the possible mechanisms, but further investigations are required, such as exploration of the relationship between splenic volume and other clinical characteristics. Furthermore, clinical stage, COPD concomitant state, and ALC are independent prognostic indicators for patients with advanced or locally advanced NSCLC treated with chemoradiotherapy.

## 7 Data access statement

All relevant data are within the paper and its Supporting Information files.

## Author contributions

**Conceptualization:** Jianping Guo, Jiandong Zhang.

**Data curation:** Jianping Guo, Yajuan Lü, Chong Hao.

**Formal analysis:** Lei Wang, Xiaoyan Wang.

**Funding acquisition:** Jiandong Zhang.

**Investigation:** Luo Li, Congcong Wang.

**Resources:** Jianping Guo, Jiandong Zhang.

**Software:** Lei Wang, Xiaoyan Wang.

**Supervision:** Yajuan Lü.

**Validation:** Luo Li, Congcong Wang.

**Visualization:** Chong Hao.

**Writing – original draft:** Jianping Guo, jiandong Zhang.

**Writing – review & editing:** Jianping Guo, jiandong Zhang.
